# Heavy metal concentrations in feathers and metabolomic profiles in Pacific black ducks (*Anas superciliosa*) from Southeastern Australia

**DOI:** 10.1093/etojnl/vgae004

**Published:** 2025-01-06

**Authors:** Damien Nzabanita, Hao Shen, Stephen Grist, Jordan O Hampton, Jasmin Hufschmid, Dayanthi Nugegoda

**Affiliations:** School of Science, Royal Melbourne Institute of Technology, Melbourne, Victoria, Australia; School of Science, Royal Melbourne Institute of Technology, Melbourne, Victoria, Australia; School of Science, Royal Melbourne Institute of Technology, Melbourne, Victoria, Australia; Faculty of Science, University of Melbourne, Melbourne, Victoria, Australia; School of Veterinary Medicine, Murdoch University, Murdoch, Western Australia, Australia; Faculty of Science, University of Melbourne, Melbourne, Victoria, Australia; School of Science, Royal Melbourne Institute of Technology, Melbourne, Victoria, Australia

**Keywords:** biomonitoring, ducks, ecotoxicology, heavy metals, metabolome

## Abstract

Heavy metals are cumulative toxicants that frequently create negative health effects for waterbirds inhibiting contaminated freshwater systems. Although levels of exposure to heavy metals have been well documented for many waterbird species, the adverse effects of exposure remain relatively poorly understood. One emerging field that allows the exploration of such effects is metabolomics. The aim of this study was to characterize metabolomic profiles in relation to long-term heavy metal exposure in a waterbird species. In 2021, wings from 44 Pacific black ducks (*Anas superciliosa*) were collected by recreational hunters at three sites in Victoria, southeastern Australia. The concentrations of seven heavy metals were measured in feathers and these data were quantified via inductive coupled plasma mass spectrometry and compared with a semiquantitative assessment of 21 metabolites identified in muscle tissues from the same birds via gas chromatography-mass spectrometry. Principal component analysis was conducted to test associations between metabolites, heavy metals, and sites. Mean heavy metal concentrations detected were copper (9.97 µg/g), chromium (0.73 µg/g), iron (123.24 µg/g), manganese (13.01 µg/g), mercury (0.58 µg/g), lead (0.86 µg/g), and zinc (183.95 µg/g; dry wt). No association was found between heavy metals and 17 metabolites, whereas four metabolites were negatively associated with some heavy metals: α-linolenic acid with iron, glucose with lead and manganese, lactic acid with mercury, and propanoic acid with mercury. There were few differences in the studied metabolites in ducks between the three sites. This study provides a novel approach to combining toxicological and metabolomic data for an ecologically important species from a relatively poorly studied global region.

## Introduction

Waterbirds face an imperiled future globally (Amano et al., 2020), and particularly in Australia (Mott et al., 2023). Anthropogenic threats to their populations include climate change, human water use, hunting, introduced animal species, infectious diseases, and harmful chemical pollutants (Kingsford, 2013). Growing awareness of the importance of pollutants for these taxa has seen the field of ecotoxicology become an increasingly important focus of wildlife health discussions worldwide, but its importance has been underrecognized in Australia until recently (Death et al., 2019). For many harmful chemicals, the extent of exposure and the effects on the health of wildlife remain unknown. One group of toxicants for which there has been considerable global research is the heavy metals (Solgi et al., 2020; Varagiya et al., 2022).

Environmental pollution with heavy metals, such as lead, mercury, and chromium, has long been recognized as a global threat to aquatic ecosystems (Ali et al., 2019). Australian studies that have examined heavy metal exposure in wildlife have focused on species as diverse as seabirds, for example., little penguins (*Eudyptula minor*; Finger et al., 2015), arboreal mammals, for example, fruit bats (Sánchez et al., 2022; Skerratt et al., 1998), and urban reptiles, for example, venomous snakes (Lettoof et al., 2021). Waterbirds are also exposed to these toxicants, resulting in sporadic mass mortality events (Koh & Harper, 1988) as well as numerous more subtle negative health effects (Zhang & Ma, 2011). In addition to being directly affected by these chemicals, waterbird species are regarded as useful indicators (“bioindicators”) of environmental health in freshwater systems (Amat & Green, 2010), because they often feed in the upper trophic levels of aquatic food webs. Given the ecological importance and susceptibility to population decline of Australian waterbirds (Kingsford & Thomas, 1995), there is a need to understand how differing levels of exposure to heavy metals may affect their physiology. One promising approach to achieving this goal is metabolomics (Liu et al., 2022).

Metabolomics refers to the systematic study of changes in low molecular weight metabolites (lipids, sugars, and amino acids) within an organism in response to endogenous and exogenous stressors (Hollywood et al., 2006). Metabolomic studies have been applied extensively to a variety of invertebrate (Shen et al., 2022, 2023a, 2023b), fish (Marie & Gallet, 2022), and mammal species, including humans (Wandro et al., 2017). However, until recently, there have been few studies applying metabolomics to reptile (Chang et al., 2022) or avian species and even fewer focused on waterbirds (Dorr et al., 2019; Ma et al., 2020; Perera et al., 2022; Sarma et al., 2022). Metabolomics has been successfully used to study the effects of different external stressors, including infectious disease or nutritional imbalances (Lankadurai et al., 2013). In recent years, metabolomics has increasingly been used to explore metabolic changes occurring in response to the presence of chemical pollutants, including toxicants such as heavy metals (Carmen, 2022). When combined with heavy metal analysis, metabolomics can identify the metabolic responses of an organism to the toxicological effects of the trace element (Liu et al., 2022).

One impediment to applying this promising new technological advance to Australian waterbirds is the dearth of recent studies of heavy metal exposure in these taxa (Nzabanita et al., 2023). Most studies relate to efforts to estimate lead exposure during the era when the use of lead shot was legally allowed for hunting (Kingsford et al., 1989; Koh & Harper, 1988; Olsen, 1984; Wickson et al., 1992). All such studies relied on lethal sampling to access animal tissues such as bone and liver. The use of nondestructive and noninvasive biomarkers such as feathers, however, has been shown to be a valuable alternative for the estimation of levels of exposure to heavy metals (Lodenius & Solonen, 2013; Jaspers et al., 2019; He et al., 2020; Anbazhagan et al., 2021; Morrissey et al., 2024). This has been particularly useful for studying raptors (Lodenius & Solonen, 2013) and waterbirds (Jiménez et al., 2020) and is now recognized as a reliable method for assessing long-term exposure trends (Varagiya et al., 2022) in avian species.

Feathers have been used extensively as nondestructive, noninvasive samples for monitoring heavy metal pollution (Veerle et al., 2004; Anbazhagan et al., 2021). Feather metal concentrations have been widely reported for comparative purposes of body burdens of trace metals in birds worldwide (Bianchi et al., 2008; Kim & Oh, 2014; Solgi et al., 2020). The use of feathers for this purpose is predicated on the deposition of metals during the period of feather growth (Veerle et al., 2004). However, the use of adult feathers for diagnostic purposes can be problematic because they are a marker of accumulation over a long period of time, unlike tissues such as blood, liver, and bone. For this reason, feather concentrations are less amenable to clinical categorization of short-term exposure as background or poisoning (subclinical, clinical, or severe; Franson & Pain, 2011).

In this study, we aimed to (a) examine the body burden of seven heavy metals in ducks from three freshwater biomes in southeastern Australia via feather analysis; and (b) assess whether concentrations of these heavy metals in feathers were correlated to semiquantitative analysis of metabolites in muscle tissue.

## Materials and methods

### Study species

We studied Pacific black ducks (*Anas superciliosa*), one of the most widespread and abundant duck species in Oceania. The species is found throughout Australia and their range extends into New Guinea, Indonesia, and New Zealand (Yu and Klaassen, 2022). They inhabit Australian environments ranging from coastal wetlands (Norman, 1976) to ephemeral desert lakes (McEvoy et al., 2017). This flexibility in habit usage is facilitated by their ability to undertake long-distance movements (McEvoy et al., 2015). They are one of Australia’s largest dabbling ducks, with live weight approximately 1.0 kg and limited sexual dimorphism. They molt annually and feed mainly on aquatic plants and small aquatic mollusks, crustaceans, and insects (Norman 1983). Pacific black ducks are also a valued wildlife resource for recreational hunters (Moloney et al., 2022) and consequently are amenable to opportunistic lethal sampling, leading to their use as sentinel species for several studies examining environmental contamination (Nzabanita et al., 2023) and (Nzabanita et al., 2024a).

### Study locations and sample collection

**Figure 1.**
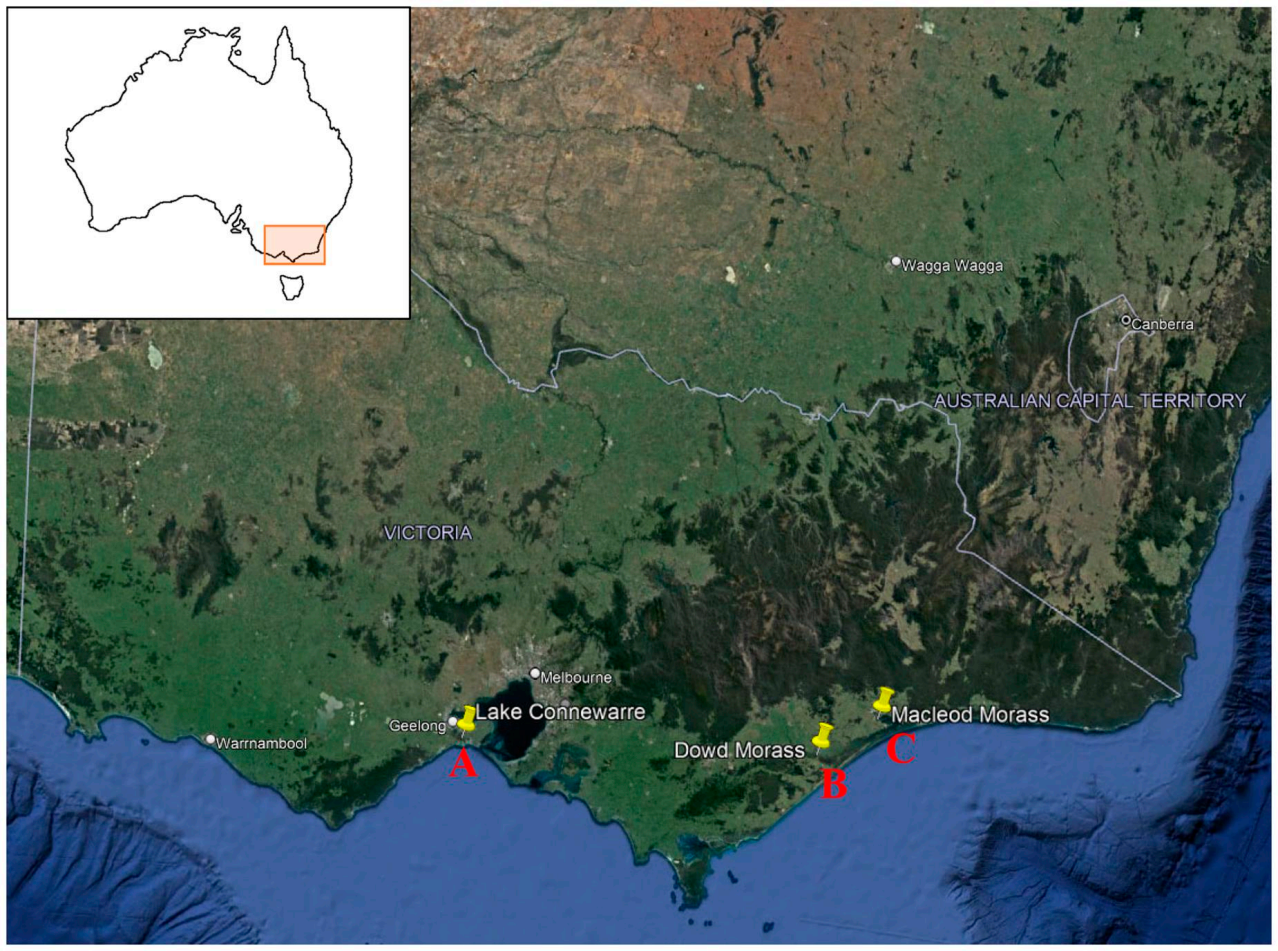
Our three field sites, Lake Connewarre (A), Dowd Morass (B), and Macleod Morass (C), where we collected wings from harvested Pacific black ducks (*Anas superciliosa*) in Victoria, southeastern mainland Australia.

We used opportunistic sampling to collect tissues from recreational duck hunters, according to Szabo et al. (2021) and Nzabanita et al. (2023). Wings from a total of 44 freshly dead Pacific black ducks were collected from three locations in southern Victoria, Australia (Figure 1): Lake Connewarre Game Reserve (38°15' S, 144°29' E; *n* = 11; all females), Dowd Morass Wildlife Reserve (38°08' S, 147°13' E; *n* = 14; six males and eight females), and Macleod Morass Wildlife Reserve (37°54' S, 147°39' E; *n* = 19; all females). All of these birds were adults—not first-year birds—as indicated by the standard process used in Victoria (Australia) for ageing and sexing Victorian native game birds via plumage characters (Rogers et al. 2019).

Samples were collected opportunistically through routine wing collection by government staff monitoring the harvest of licensed hunters in May 2021 as part of the official duck hunting season (Menkhorst et al., 2019). Pacific black ducks display annual molting, which typically occurs in December–April and is characterized by a period of enhanced energy expenditure (Yu and Klaassen, 2022). Because feathers were collected soon after this annual molting period, metals deposited in the relatively young feathers that we sampled were likely to be indicative of recent exposure. After collection, one wing from each bird was frozen (−20°C) before being dissected; after dissection, the underwing coverts and axillary feathers were plucked from the wings and placed in labelled plastic zip-lock bags until analysis. Wing muscles overlying the radius and ulna were surgically dissected from the bone; they were then put in polypropylene transport tubes and stored (−80°C) until metabolite analysis.

### Heavy metal concentrations in feathers

Feather samples were prepared and analyzed according to the method of Nzabanita et al. (2024b). Briefly, multiple feathers were washed in petri dishes using a mixed solution of chloroform (CHCl_3_) and methanol (CH_3_OH) to remove external contaminants. Then, the samples were carefully rinsed three times with deionized water (H_2_O) to remove the solvent and air-dried for 24 hr (Liu et al., 2019). The dried feathers were cut into small pieces as close to the rachis (shaft) as possible, then between 15 and 20 mg of sample was added to acid-washed polypropylene test tubes for digestion.

Nitric acid (HNO_3_) 70% was added to each test tube and capped with a glass marble ball to prevent sublimation and evaporation. The mixture was placed on heating blocks for 24 hr until the solution was completely digested and clear. Afterwards, all samples were diluted and transferred to centrifuge tubes at 5°C until the time of analysis by inductive coupled plasma mass spectrometry (ICP-MS). To prevent any undissolved sample solution from affecting the ICP-MS, a 0.45 μm disposable filter was used. After filtration, a hydrochloric acid solution was transferred to each of the Falcon conical centrifuge tubes (Falcon Tubes, Siliguri, India) for subsequent ICP-MS analysis. A 7700X ICP-MS machine (Agilent Technologies, Santa Clara, USA) was used to analyze the concentrations of chromium, copper, iron, mercury, manganese, lead, and zinc. For quality control, human hair (trace elements) standard reference materials and Measurements ERM-DB001 (European Virtual Institute for Speciation Analysis) were used to determine the experimental recovery efficiency. The heavy metals used and reported in this study for the quality control had recovery rates of 80%–120%.

### Metabolomics analysis from muscle samples

We used semi-quantitative gas chromatography-mass spectrometry (GC-MS)-based untargeted metabolomics analysis, as described by Shen et al. (2022, 2023a, 2023b). Briefly, each muscle sample was thawed, weighed (0.1 ± 0.01 g) and refrozen in liquid nitrogen (−20°C); tissue was then homogenized with a 1 mL ratio (1:3:1) mixture of CHCl_3_/CH_3_OH/H_2_O. Homogenized samples were then vortexed for 1 minute and incubated for 1 hr at −20°C. They were then centrifuged for 25 minutes at 5,000 rpm in a SIGMA 3-16KL refrigerated centrifuge (Sigma Laborzentrifugen GmbH, Osterode am Harz, Germany), and supernatants were extracted and stored at −80°C.

We transferred 100 µL of the extracted supernatant samples to 1.5 mL screw neck glass vials with micro-inserts. Samples were dried at room temperature in nitrogen steam for 1 hr. Subsequently, 40 µL methoxyamine hydrochloride in pyridine 20 mg/mL (w/v) was added to each sample, after which they were vortexed for 15 s and incubated at 35°C for 1 hr. This process was followed by the addition of 60 µL of N-methyl-N-trimethylsilyl trifluoroacetamide + 1% trimethylchlorosilane N-methyl-N-trimethylsilyl tri fluoroacetamide, and samples were then vortexed for 30 s to prepare for GC-MS analysis. Chromatogram results with retention time and each peak of the sample were analyzed individually. For quality control, CRM18918 F.A.M. E. C8–C24 mixture, certified reference material, and AA-S-18 amino acid standard solution were analyzed under similar instrument conditions and their retention times and mass spectra were used as matches for the identification of metabolites (Shen et al., 2022).

The raw data on the metabolites were analyzed using Agilent Mass-Hunter Qualitative Analysis Navigator (B.08.00) and the peaks were integrated for area matrices and identified using the National Institue of Standards and Technology 20 Mass Spectral library (Shen et al., 2022). Each metabolite was scored as present or absent from each analyzed sample and relative abundance was calculated (Lee et al., 2019). Metabolites that were significantly changed were confirmed based on the intensities of their mass-to-charge (*m/z*) features and area matrices (Shen et al., 2022).

### Statistical analysis

The IBM software SPSS Statistics, Ver. 28, was used to analyze the metals data from the feathers. Differences in mean trace metal concentration between the three sites were investigated using a one-way analysis of variance (ANOVA) test, and the significance level was set at *p *<0.05. For pair-wise comparison of differences between specific areas, post hoc testing using Student *t*-tests with Bonferroni correction was applied. An independent sample *t*-test was used to determine whether there was a significant difference between males and females from the only site that included male animals, Dowd Morass (all samples from other sites were from females).

The MetaboAnalyst (V5.0) platform was used to analyze all annotated metabolites (Shen et al., 2022). Rstudio 3.6.0, GGPLOT2 3.1.0, FactoMineR 1.41, and Factoextra 1.0.5 data analysis software were used to generate principal component analysis (PCA) biplots (Shen et al., 2023a). Principal component analysis was used to examine whether any muscle metabolite levels were correlated to feather heavy metal levels, taking into consideration location and sex. Area matrices and *m/z* features were used to analyze differences in relative abundance of metabolites between sites, and the results were visualized as a score plot, according to Shen et al. (2022). We then ran a one-way ANOVA, according to Shen et al. (2023a), to examine whether any specific metabolite changed among sites. All results are given as mean ± standard error unless otherwise stated.

## Results

### Heavy metal levels

**Figure 2.**
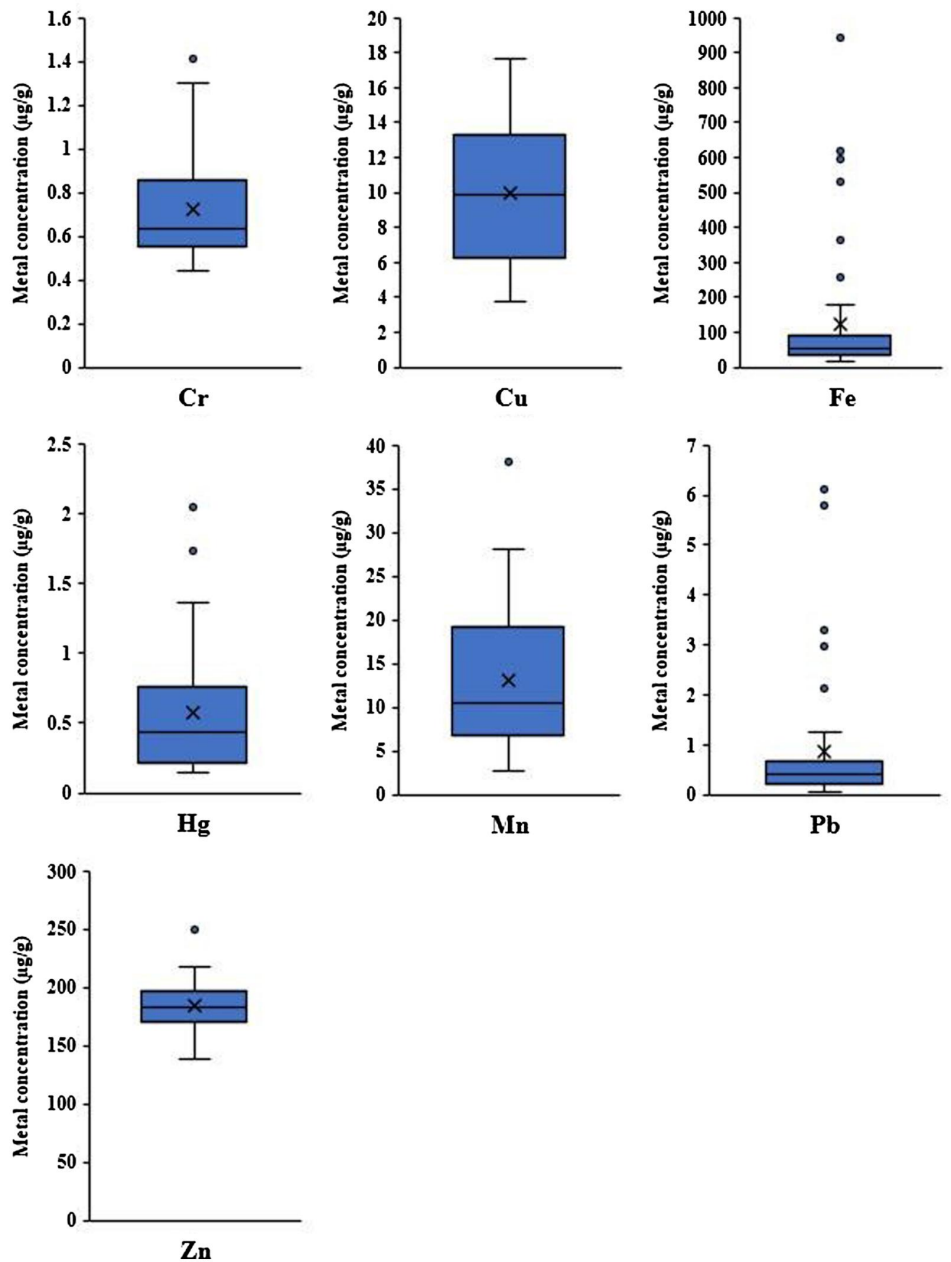
Box and whisker plots showing the distribution of concentrations (µg/g dry wt) of seven heavy metals in feathers from Pacific black ducks (*Anas superciliosa*) sampled from southeastern Australia in 2021. The tail whiskers represent the minimum and maximum metal concentrations; the lower and upper horizontal bars on the boxes indicate the metal concentrations of quartiles one and three (Q1 and Q3) whereas the median concentrations are horizontal lines in the boxes. The cross (×) symbols are the mean concentrations and the circles (•) represent the outlier metal concentrations.

The results of the concentrations of heavy metals in feathers are shown in Figure 2. Mean concentrations detected were copper (9.97 ± 0.58 µg/g), chromium (0.73 ± 0.04 µg/g), iron (123.24 ± 29.07 µg/g), manganese (13.01 ± 1.22 µg/g), mercury (0.58 ± 0.08 µg/g), lead (0.86 ± 0.20 µg/g), and zinc (183.95 ± 3.08 µg/g; dry wt). For details, see online supplementary material Table S1.

**Figure 3.**
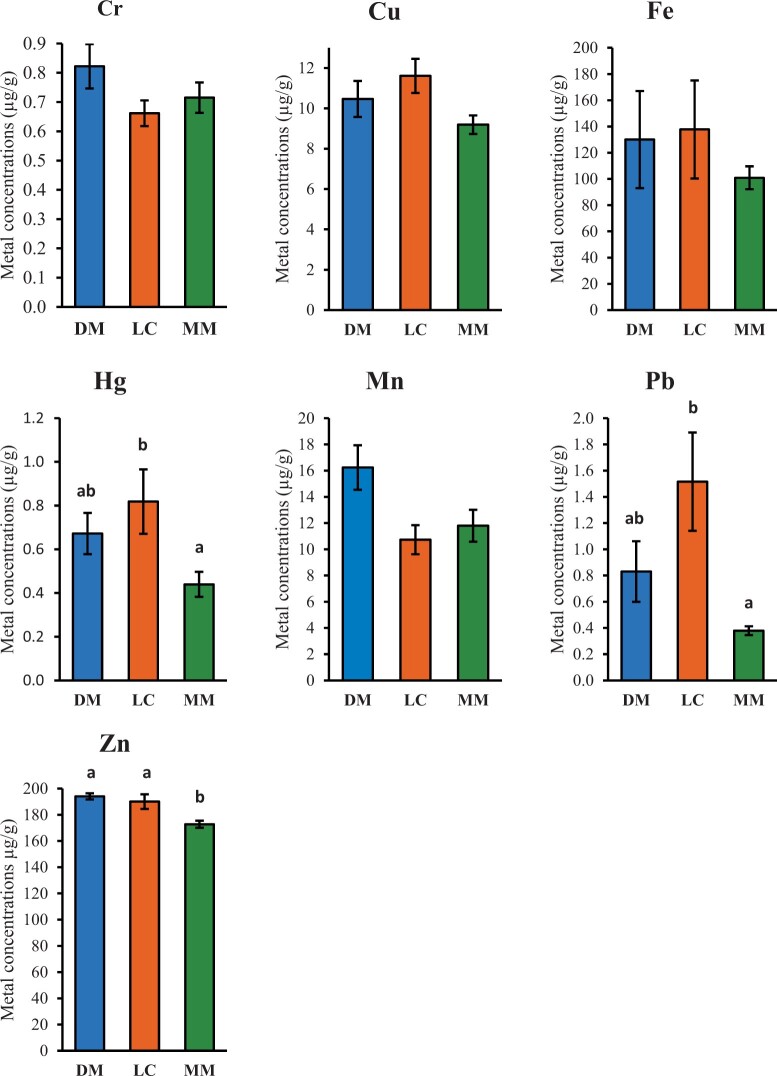
Mean concentrations (µg/g dry wt) of heavy metals in the wing feathers of female Pacific black ducks (*Anas superciliosa*) sampled in southeastern Australia in 2021 by location. DM = Dowd Morass; LC = Lake Connewarre; MM = Macleod Morass (see Figure 1). Error bars indicate the standard error of the mean. The letters ‘a’, ‘b’ and ‘ab’ above columns depict significant differences, whereas the absence of letters above columns indicates that there were no significant differences between sites for this element.

The mean concentration of most metals did not differ significantly between sites (Figure 3). However, lead (the magnitude of the mean difference = 1.15; 95% confidence interval [CI], 0.134 to 2.174; F(2,36) = 4.043; *p = *.022) and mercury (mean difference = 0.45; 95% CI, 0.003 to 0.894; F(2,36) = 4.185; *p *=* *0.048) levels in feathers were significantly higher at Lake Connewarre (the peri-urban site) than Macleod Morass (a rural site). Compared with Macleod Morass, these metals also trended towards being higher in feathers from the birds at the other rural site, Dowd Morass, although not statistically significant. Zinc levels were lower in feathers from birds at Macleod Morass than at either Lake Connewarre or Dowd Morass (mean difference = 21.20; 95% CI, −40.844 to −1.572; F(2,36) = 4.836; *p = *0.031).

Sex variation was analyzed for birds at Dowd Morass only (it was the sole site at which we had samples from both sexes). There was no statistically significant difference in heavy metal concentrations between sexes. However, to enable between-site comparisons, trace metal concentrations in feathers and metabolites were reported only for female ducks.

### Metabolome profile and correlations with heavy metals

**Figure 4.**
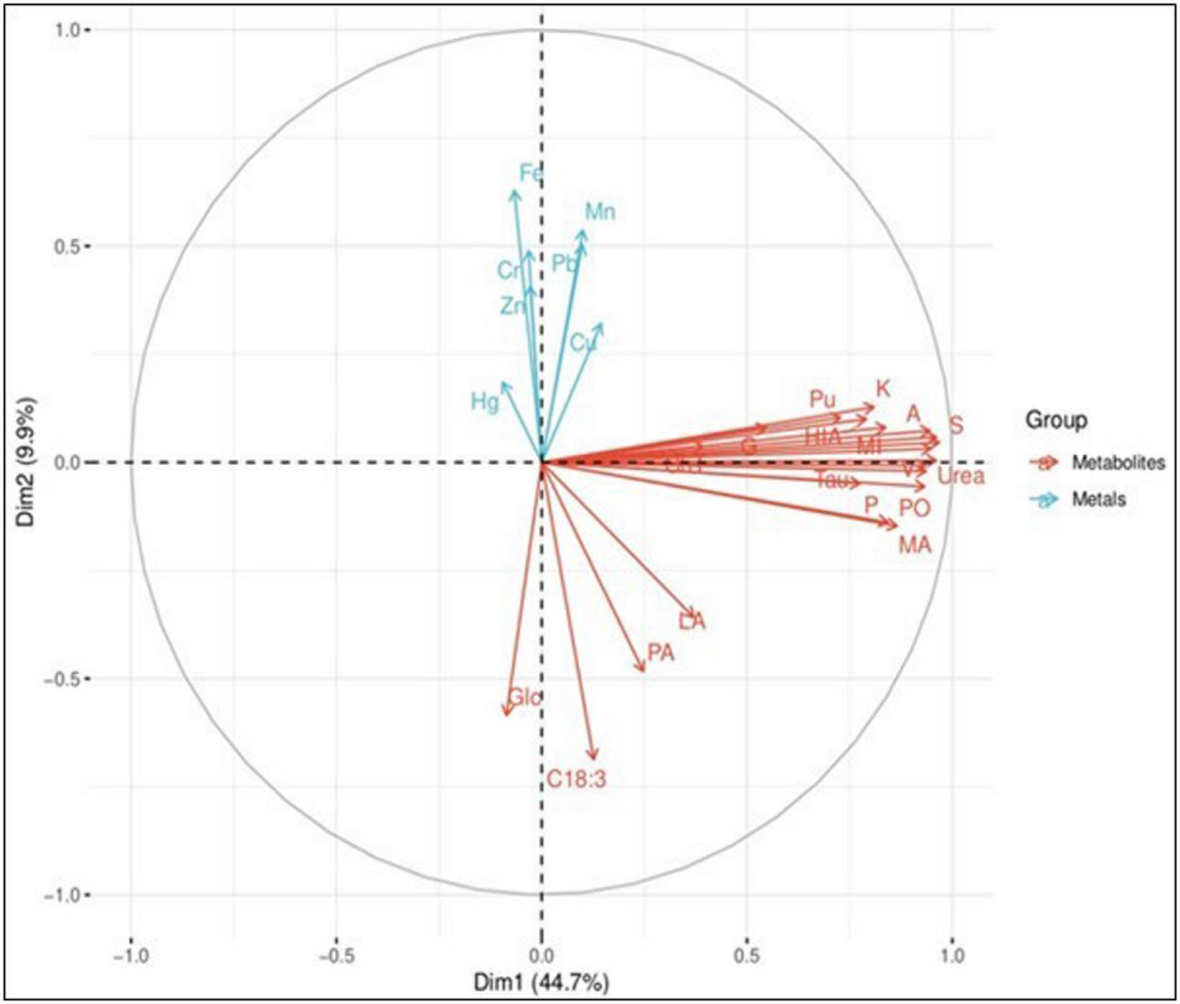
Principal component analysis biplot of seven heavy metal concentrations in feathers (blue arrows) and 21 metabolites in muscle tissue (orange arrows) from Pacific black ducks (*Anas superciliosa*) sampled from three different sites (pooled) in southeastern Australia in 2021. LA = lactic acid; A = L-alanine; D = L-aspartic acid; L = L-leucine; K = L-lysine; P = L-proline; V = L-valine; T = N.O.O L-threonine; G = glycine; S = serine; HIA = 1H-imidazole-2-amine; Glc = D-glucose; MI = myo-inositol; Tau = taurine; MA = malic acid; PA = propanoic acid; ChT = cholesterol; Pu = 9H-purine; PO = phosphate; C18:3 = α-linolenic acid.

**Figure 5.**
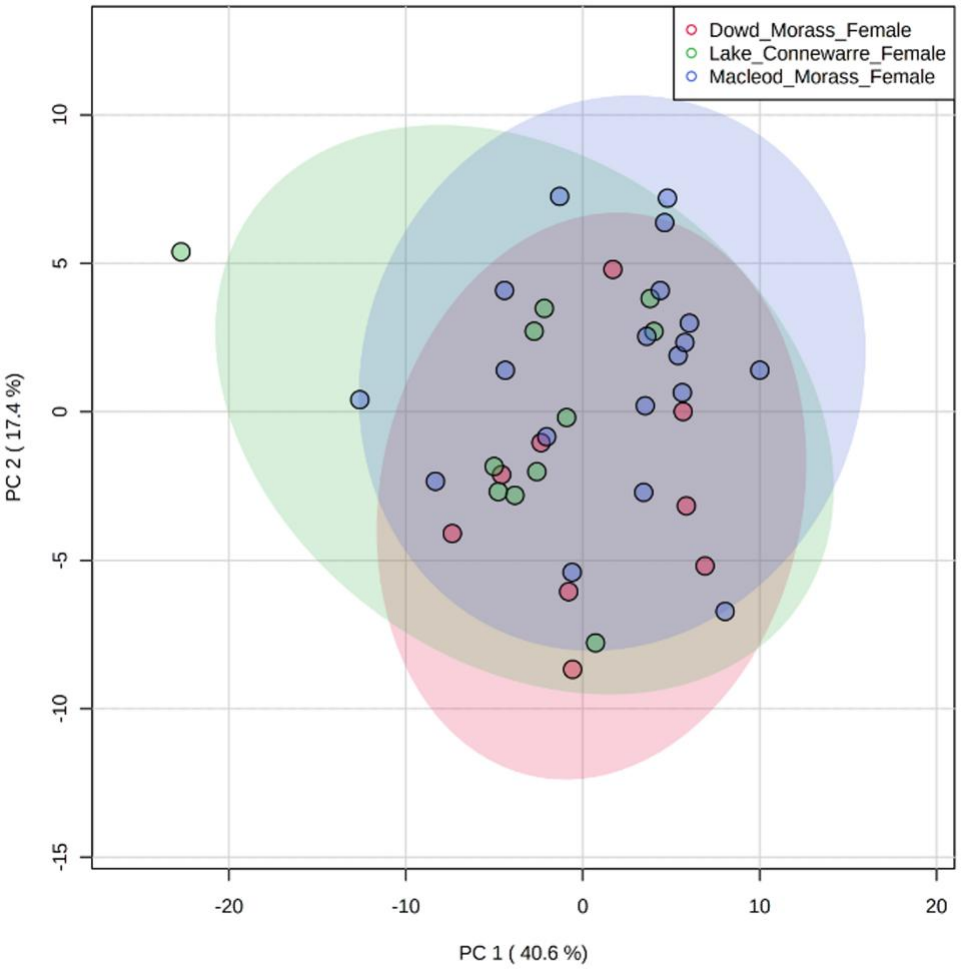
Principal component analysis (PCA) score plot showing differences in metabolites in muscle tissue from female Pacific black ducks (*Anas superciliosa*) sampled from three different sites: Dowd Morass (*n =  *8), Lake Connewarre (*n = *11), and Macleod Morass (*n = *19) from southeastern Australia in 2021.

In total, 41 metabolites were identified from wing muscle samples taken from female ducks. These constituted amino acids, sugars, organic acids, free fatty acids, urea, phosphate, 9H-purine, and cholesterol; for details, see online supplementary material Table S2. Out of these 41, only 21 metabolites (51%) were “annotated” or could be confidently assigned. The PCA score plots (Figure 4) showed that all annotated metabolites had positive values in the first component (Dim 1) axis. Overall, there were no significant associations between heavy metal and metabolite concentrations. Of the 21 annotated metabolites, 17 (81%) were not associated with heavy metal concentrations, whereas four metabolites (19%) were negatively associated with some heavy metal concentrations: α-linolenic acid with iron, glucose with lead and manganese, lactic acid with mercury, and propanoic acid with mercury (Figure 4). The variation of metabolites within sites was performed using *m/z* characteristics according to Shen et al. (2023a). Overall, there were few differences in the studied metabolites between the three sites. The distribution of PCA score plots showed that metabolite clusters were not clearly separated, and they were significantly overlapped among sites (Figure 5). A one-way ANOVA showed no variation in metabolite concentrations between sites (*p* > 0.05) except for five metabolites (α-linolenic acid, lactic acid, aminoimidazole, taurine, and L-lysine), see online supplementary material Table S3.

## Discussion

This study provided proof of concept that metabolomic biomarkers can be useful when analyzed in combination with heavy metal body burdens as reflected in the feathers of waterbirds. To our knowledge, this is the first study to apply metabolomics to Australian waterbirds, although a similar approach was taken by Perera et al. (2022) for a Northern Hemisphere duck species, the lesser scaup (*Aythya affinis*). There was no overall correlation between heavy metal exposure levels and metabolite profiles in the animals we studied. We found that levels of lead and mercury were elevated in ducks from a peri-urban site when compared with rural sites. Our study presents a novel approach to combining toxicological and metabolomic data for an ecologically important species from a relatively poorly studied global region.

Metal concentrations of chromium, copper, manganese, and mercury displayed greater variability than did iron, lead, and zinc concentrations (Figure 2). Our results are broadly comparable to studies conducted in past decades in Australia and to similar studies globally (Table 1). However, many of the studies cited in Table 1 were performed in past decades, and there have been profound changes to the abundances and habitats of these species in Australia since that time (Brandis et al., 2021).

**Table 1.** Concentrations of heavy metals (mean ± SEM, µg/g dry wt) in feathers of ducks and geese species reported in the published literature.

Species	Location	Cr	Cu	Fe	Hg	Mn	Pb	Zn	Citation
**Pacific black duck (*Anas superciliosa*)**	South-eastern Australia	0.7 ± 0.0	10.0 ± 0.6	123.2 ± 29.1	0.6 ± 0.1	13.0 ± 1.2	0.9 ± 0.2	183.9 ± 3.1	This study
**Pacific black duck**	South-eastern Australia	–	–	–	–	–	6.0 ± 14.3	–	Wickson et al. 1992
**Pacific black duck**	Northern Australia	6.2 ± 1.8	12.0 ± 0.92	–	–	11.0 ± 4.1	1.5 ± 0.5	190.0 ± 23.0	Brennan et al. 1992
**Greylag goose (*Anser anser*)**	South-western France	–	35.7 ± 41.0	–	0.1 ± 0.0	–	9.3 ± 8.2	100.6 ± 28.3	Lucia et al. 2010
**Canada goose (*Branta canadensis*)**	Northeastern USA	1.4 ± 0.2	–	–	0.3 ± 0.0	–	1.9 ± 0.4	–	Tsipoura et al. 2011
**Ferruginous duck (*Aythya nyroca*)**	Northern China	0.8 ± 0.1	13.8 ± 1.0	410.9 ± 84.0	0.3 ± 0.1	11.2 ± 2.0	2.2 ± 0.4	107.1 ± 2.5	Liu et al. 2019
**Mallard (*Anas platyrhynchos*)**	Northern Iran	–	14.1 ± 4.1	–	–	–	0.9 ± 0.5	47.3 ± 1.0	Solgi et al. 2020
**Mallard**	Northwestern South Korea	1.6 ± 0.4	12.4 ± 1.8	531.0 ± 251.0	–	27.0 ± 2.8	6.3 ± 1.4	70.2 ± 8.5	Kim and Oh 2014

The elevated levels of mercury and lead observed at the Lake Connewarre site were not unexpected, given the proximity of the lake to the city of Greater Geelong, the second largest city in Victoria (Li et al., 2020), which has an adjacent oil refinery (Dong et al., 2020). In comparison, feathers derived from two rural areas showed markedly lower heavy metal exposure. It is likely that heavy metals observed at the Lake Connewarre site are derived from the nearby city of Geelong or the Barwon River. In 2015, elevated levels of mercury and lead were reported in sediments from Lake Connewarre, with mercury concentrations exceeding the Australian Interim Sediment Quality Guideline in sediments collected from 0–30 cm depth (Reeves et al., 2015). This suggests that the significantly higher mercury burdens observed at this site are associated with higher levels of mercury pollution in this area.

The mercury concentrations we report are concerning for their potential effects on bird health (Berger et al., 2019; Boening, 2000). For context, the mean concentrations of mercury we report (0.6 µg/g) are much higher than those found in the feathers of Canada geese (*Branta canadensis*; 0.3 ± 0.03 µg/g) in the USA (Tsipoura et al., 2011), ferruginous ducks (*Aythya nyroca*; 0.3 ± 0.1 µg/g) in China (Liu et al., 2019), or greylag geese (*Anser anser*; 0.1 ± 0.0 µg/g) in France (Lucia et al., 2010), as summarized in Table 1. The only published study that has reported mercury levels in Australian ducks was the study of Olsen (1984), which examined concentrations in breast muscle of Pacific black ducks from a site in inland eastern Australia. Mercury pollution is very much also a concern for human health (Tchounwou et al., 2003), although this threat has tended to be largely overlooked in Australia (Schneider, 2021). Because Pacific black ducks in Victoria are commonly consumed by recreational hunters (Sharp et al., 2021), the levels of mercury exposure we report are concerning for their potential effects on the health of human consumers of game meat. However, we measured mercury concentrations in feathers, rather than edible meat (muscle), because we would have if our focus was on human health, as according to Sharp et al. (2021).

Since the 1990s, there have been few studies of heavy metal bioaccumulation in Victorian Pacific black ducks, or any other duck species that feed in these areas, to draw comparisons with. In the Northern Territory of Australia, a much more sparsely populated region, Brennan et al. (1992) reported the concentrations of some essential trace elements in various Australian waterbird tissues, including feathers of Pacific black ducks. They reported lead levels of 1.5 ± 0.5 µg/g dry weight in feathers, similar to lead levels in the samples analyzed in our study from Lake Connewarre (Figure 3), and higher than at the rural sites we examined.

This study was restricted to being a preliminary investigation, and essentially provided only proof-of-concept for the utility of incorporating metabolomics into Australian ecotoxicology investigations. In most of the annotated metabolites, we found no association with heavy metal exposure. The finding that lactic acid was negatively correlated with mercury exposure (Figure 4) warrants further investigation regarding the potential deleterious health effects of mercury exposure related to muscular activity in this species. The low level of correlation between heavy metals and metabolites was possibly due to the low concentrations of heavy metals detected. For reference, the normally cited level of concern for mercury in feathers is 5 mg/kg, whereas for lead, it is 4 mg/kg, and none of our samples exceeded these levels (Burger et al., 2009).

The differences between our sites in levels of five metabolites were not correlated with heavy metal levels but may instead have been influenced by exposure to persistent organic pollutants or other contaminants. Recently, considerable levels of polycyclic aromatic hydrocarbons and minor exposure to organochlorine pesticides and polychlorinated biphenyls was detected in another common Australian duck species, the grey teal (*Anas gracilis*; Nzabanita et al., 2024a). Therefore, it would be interesting for future studies to assess associations between persistent organic pollutants and metabolites in this species at these sites.

Our study had several limitations. First, our sample size was relatively small (*n* = 44), so our ability to generalize regarding contaminant exposure was limited. Second, we collected samples from a single duck species from a relatively small geographical area and from a single year. Third, our samples were sex-biased due to recreational hunting selection, with only one site having both male and female samples, which could have led to some bias in inference. Fourth, we used only feathers as our tissue type, limiting our ability to report the proportion of birds sampled that had clinical vs. background exposure to harmful heavy metals, namely lead and mercury. Fifth, our metabolite analysis was semiquantitative (Shen et al., 2022), restricting our ability to fully characterize the relationship between metabolites and heavy metals. This is typical of mass spectrometry–based metabolomics data. Although the signal itself (metabolite peak area) does not reflect the absolute concentration, differences in peak area do scale linearly with metabolite concentration (Liu & Locasale, 2017). Because our study was based on the semiquantification of metabolites (presence/absence and relative abundance; Lee et al., 2019), we cannot confirm that there is a direct effect of mercury on lactic acid and propanoic acids because we were unable to quantify the concentrations of these metabolites. Sixth, we used an untargeted strategy for selecting metabolites (Schrimpe-Rutledge et al., 2016), which contributed to the fourth limitation listed above. Finally, it was difficult to compare our metabolomic results with previous studies because there have been relatively few studies to examine the correlation between heavy metals and metabolites in birds (Sarma et al., 2022) and even fewer in ducks (Perera et al., 2022). Nonetheless, our results suggest that metabolomics could be a useful addition to the ecotoxicological tool kit applied to monitor the health of Australian wildlife species.

## Conclusion

It has been argued that Australia has a history of overlooking heavy metal contamination that can affect environmental and human health. We found that current levels of heavy metal exposure in Australian Pacific black ducks were comparable (or lower in the case of lead) to those of previous studies on the same species. However, the mercury burden we reported is of some concern, being considerably higher than for other waterbird species studied globally. In addition, the highest concentrations of mercury that we detected were at a site with a known history of mercury contamination and were hence not unexpected. Gas chromatography-MS-based untargeted metabolomics has been proposed as a valuable part of trace metal pollution evaluation and ecosystem health monitoring (Shen et al., 2022). However, this untargeted approach revealed that only 19% of all metabolites detected were associated with heavy metals. As such, we acknowledge that our results are preliminary but can be valuable to access for future studies. Given that these relationships have never been reported in Australia before, these data provide encouragement for future research in this field. These results will help to characterize the baseline metabolomic profiles of Australasian waterbird species with different trace element body burdens. We encourage future studies to build on this preliminary investigation to quantify targeted metabolites in relation to contaminants of concern in wildlife species for which conservation or public health risks are pronounced.

## Supplementary Material

vgae004_Supplementary_Data

## Data Availability

Data available on request from the corresponding author (s3362989@student.rmit.edu.au).
