# Supplementary material for: Heavy metal concentrations in feathers and metabolomic profiles in Pacific black ducks (Anas superciliosa) from Southeastern Australia
Source: Environ Toxicol Chem. 2025 Jan 6;44(1):92–102. doi: 10.1093/etojnl/vgae004 (PMC11790206; doi:10.1093/etojnl/vgae004)
Supplement: vgae004_Supplementary_Data [file vgae004_supplementary_data.docx]

**Supporting Information**

**Heavy metal concentrations in feathers and metabolomic profiles in pacific black ducks (*Anas superciliosa*) from south-eastern Australia**

**Damien Nzabanita^1*^ ● Hao Shen^1^ ● Stephen Grist^1^ ● Jordan O. Hampton^2,3^ ● Jasmin Hufschmid^2^ ● Dayanthi Nugegoda^1^**

^1^ School of Science, Royal Melbourne Institute of Technology, Melbourne Victoria 3083, Australia

^2^ Faculty of Science, The University of Melbourne, Werribee Victoria 3030, Australia.

^3^ School of Veterinary Medicine, Murdoch University, Murdoch, Western Australia 6150, Australia

**SUPPLEMENTARY** **TABLES**

**TABLE S1.** Heavy metal concentrations detected in feathers from Pacific black ducks (*Anas superciliosa*) sampled from southeastern Australia in 2021. Q1 = first quartiles of metals, Q3 = third quartiles, IQR = interquartile range, SD = standard deviations, SEM = standard error of the mean.

|  | **Cr** | **Cu** | **Fe** | **Hg** | **Mn** | **Pb** | **Zn** |
| --- | --- | --- | --- | --- | --- | --- | --- |
| Minimum | 0.44 | 3.74 | 14.83 | 0.15 | 2.70 | 0.05 | 139.00 |
| Maximum | 1.41 | 17.68 | 177.42 | 2.05 | 38.13 | 6.13 | 249.03 |
| Mean | 0.73 | 9.97 | 123.24 | 0.58 | 13.01 | 0.86 | 183.95 |
| Q1 | 0.55 | 6.22 | 33.96 | 0.22 | 6.83 | 0.21 | 169.94 |
| Q3 | 0.86 | 13.28 | 92.78 | 0.76 | 19.27 | 0.67 | 196.48 |
| Median | 0.63 | 9.92 | 52.82 | 0.47 | 10.42 | 0.41 | 182.24 |
| SD | 0.25 | 3.83 | 192.80 | 0.46 | 8.09 | 1.33 | 20.40 |
| SEM | 0.04 | 0.58 | 29.07 | 0.08 | 1.22 | 0.20 | 3.08 |
| Range | 0.97 | 13.94 | 928.98 | 1.90 | 35.42 | 6.08 | 110.04 |
| IQR | 0.31 | 7.06 | 58.82 | 0.53 | 12.44 | 0.46 | 26.54 |

**TABLE S2.** All Classes identified metabolites and their retention times (RT). Star symbols (*****) depict the annotated metabolites identified in wing muscle of Pacific black ducks (*Anas superciliosa*) sampled from southeastern Australia in 2021.

| **Classification** | **Identifies untargeted metabolites** | | **Retention time (minutes)** | | **Annotated metabolites** | |  |
| --- | --- | --- | --- | --- | --- | --- | --- |
| **Amino acid** | Lactic acid | | 5.525 | | * | |  |
|  | L-alanine | | 6.201 | | * | |  |
|  | Glycine | | 6.512 | | * | |  |
|  | L-valine | | 7.922 | | * | |  |
|  | L-leucine | | 9.045 | | * | |  |
|  | L-isoleucine | | 9.108 | | - | |  |
|  | L-proline | | 9.224 | | * | |  |
|  | Serine | | 9.972 | | * | |  |
|  | N.O.O L-threonine | | 10.31 | | * | |  |
|  | L-Aspartic acid | | 12.05 | | * | |  |
|  | Cysteine | | 12.538 | | - | |  |
|  | Glutamic acid | | 13.249 | | - | |  |
|  | N.O. Phenylalanine | | 13.327 | | - | |  |
|  | 1H-imidazol-2-amine | | 12.446 | | * | |  |
|  | L-Lysine | | 15.811 | | * | |  |
|  | L-tyrosine | | 16.678 | | - | |  |
|  | Inosine | | 22.203 | | - | |  |
| **Sugar** | D-Glucose | | 16.226 | | * | |  |
|  | Inositol | | 17.516 | | - | |  |
|  | Glucopyranose | | 17.044 | | - | |  |
|  | Myo-Inositol | | 18.112 | | * | |  |
| **Organic acid** | Benzoic acid | | 7.706 | | - | |  |
|  | Propanoic acid | | 9.558 | | * | |  |
|  | Pyrimidine acid | | 9.665 | | - | |  |
|  | 2-butenedioic acid | | 9.869 | | - | |  |
|  | Malic acid | | 11.647 | | * | |  |
|  | Taurine | | 13.79 | | * | |  |
|  | D-Gluconic acid | | 17.247 | | - | |  |
|  | Deoxycholic acid | | 19.383 | | - | |  |
| **Free fatty acid** | C4:0 | | 8.244 | | - | |  |
|  | C 16:0 | | 17.758 | | - | |  |
|  | C 18:2 | | 19.268 | | - | |  |
|  | C18:1 | | 19.322 | | - | |  |
|  | C18:0 | | 19.556 | | - | |  |
|  | C20:4 Δ5,8,11,14 | | 20.609 | | - | |  |
|  | C18:3 (α-linolenic acid) | | 22.126 | | * | |  |
|  | C16:0 (2,3-bis[(trimethylsilyl)oxy] propyl ester) | | 22.345 | | - | |  |
|  | C18:0 (2,3-bis[(trimethylsilyl)oxy] propyl ester) | | 23.771 | | - | |  |
| **Others** | Urea | | 8.379 | | * | |  |
|  | Phosphate | | 8.736 | | * | |  |
|  | 9H-Purine | | 15.315 | | * | |  |
|  | Cholesterol | | 26.268 | | * | |  |
|  |  |  | |  | |  | |

**TABLE S3.** Differences in metabolite profiles in wing muscle of female Pacific black ducks (*Anas superciliosa*) sampled from southeastern Australia in 2021 by locations, based on one-way ANOVA and post-hoc 2-sample t-tests. Letters a and b depict differences in annotated mass-to-charge (*m/z*), where the same letter shows no change (*p* < 0.05). DMF = Dowd Morass female; LCF = Lake Connewarre female; MMF = MacLeod Morass female.

| **Classification** | **Metabolites** | **DMF** | **LCF** | **MMF** | ***p* value** |
| --- | --- | --- | --- | --- | --- |
| Amino acid | Latic acid | a | b | ab | 0.012 |
|  | L-lysine | ab | a | b | 0.049 |
|  | 2-Aminoimidazole | ab | a | b | 0.038 |
| Organic acid | Taurine | ab | a | b | 0.031 |
| Fatty compound | C18:3 Δ9,12,15 (α-Linolenic acid) | a | ab | b | 0.024 |

**TABLE S4.** Limits of detection and quantification for seven metals analyzed in feather samples from pacific black ducks (*Anas superciliosa*) from south-eastern Australia

| **Element** | **Limit of detection (mg/kg)** | **Limit of quantification (mg/kg)** |
| --- | --- | --- |
| **Cr** | 0.020 | 0.175 |
| **Cu** | 0.039 | 0.175 |
| **Fe** | 0.091 | 8.75 |
| **Hg** | 0.075 | 0.086 |
| **Mn** | 0.024 | 0.086 |
| **Pb** | 0.011 | 0.086 |
| **Zn** | 0.136 | 0.438 |
